# Activation of ERK Signaling via TLR11 Induces IL-12p40 Production in Peritoneal Macrophages Challenged by *Neospora caninum*

**DOI:** 10.3389/fmicb.2017.01393

**Published:** 2017-07-26

**Authors:** Xiaoxia Jin, Pengtao Gong, Xichen Zhang, Guojiang Li, Tao Zhu, Mengge Zhang, Jianhua Li

**Affiliations:** ^1^Key Laboratory of Zoonosis, Ministry of Education – College of Veterinary Medicine, Jilin University Changchun, China; ^2^Jilin Agricultural Science and Technology University Jilin, China

**Keywords:** *N*. *caninum*, cytokine, IL-12p40, ERK signaling, TLR11

## Abstract

*Neospora caninum*, an obligate intracellular protozoan parasite, can infect a large variety of vertebrate hosts including the most economically important cattle. Infection with *N*. *caninum* is a main cause of abortion in both dairy and beef cattle, which causes great economic losses worldwide. However, the mechanism of host cell infection by *N*. *caninum* has not been fully elucidated, especially in terms of inflammatory responses. In this study, the effect of TLR-ERK signaling pathway on the synthesis of pro-inflammatory interleukin-12p40 in mouse peritoneal macrophages (PMϕ) challenged by *N*. *caninum* was investigated. Our results suggested that *N*. *caninum* infection quickly activated MEK-ERK signaling via TLR11 in PMϕ. In addition, *N*. *caninum* infection also caused upregulated production of IL-12p40 by PMϕ, which was significantly reduced with the blockade of TLR11/MEK/ERK pathway, suggesting that this upregulation of IL-12 p40 was TLR11 and MEK-ERK-activation dependent.

## Introduction

*Neospora caninum*, classified in the phylum Apicomplexa, induces severe protozoosis–Neosporosis in an abundant variety of vertebrate hosts with a worldwide distribution (Dubey, 2003). *N*. *caninum* causes abortions of pregnant animals and deaths of newborns, which lead to huge economic losses in animal production. *N*. *caninum* is a major pathogen for cattle and is efficiently vertically transmitted in cattle (Dubey and Schares, 2011). Because of the significant economic impact of neosporosis, a better understanding of *Neospora*-triggered immune response is essential to elucidate the pathogenesis caused by this parasite and is useful for the development of effective vaccines and therapeutic strategies against neosporosis. Infections in many mammalian hosts are common but clinical diseases are rare, most of which present persistent latent infections. In order to survive, *N. caninum* has developed the ability to respond to changes of living environment through stage transformation. Pregnancy-induced immune-suppression, which presents reduced Th-1 type reaction and converts into Th-2 type response, may reactivate latent tissue cysts of *N. caninum* leading to placental infection and intrauterine infection. The fetus may be infected by parasites that cross the placenta, causing abortions or congenital infections, depending on the period of gestation, parasite virulence and the maternal and fetal immunity (Innes et al., 2002).

*Neospora caninum* infections induce pro-inflammatory cytokine responses, such as interleukin-12, interferon-γ, and tumor necrosis factor-α, to control the replication of parasites in host cells. Studies have shown that synthesis of interferon (IFN)-γ by T cells and NK cells, required for host resistance to *Toxoplasma gondii* and *N. caninum* infections, is driven by IL-12 (Nishikawa et al., 2002; Sher et al., 2003). Interleukin (IL)-12, mostly produced by activated dendritic cells and hematopoietic phagocytic cells (monocytes, macrophages, neutrophils), has been widely accepted as an important regulator of T helper (Th) 1 cell responses (Langrish et al., 2004). Th1-type immune responses have been demonstrated to be crucial in the control of *N. caninum* infection. It has been reported that IFN-γ and IL-12 play key roles in control of acute neosporosis in mice (Baszler et al., 1999; Nishikawa et al., 2002). Studies have showed that C57BL/10ScCr mice, which lack a functional IL-12 receptor, are highly susceptible to *N. caninum* infection (Botelho et al., 2007). MyD88^-/-^ and IL-12p40^-/-^ mice succumbed to acute infection by *N. caninum* due to uncontrolled parasite replication, which was associated with the lack of IL-12 production and delayed IFN-γ response (Mineo et al., 2009). Extracellular signal-regulated protein kinases 1/2 (ERK1/2), which is involved in the highly conserved mitogen activated protein kinase (MAPK) signal transduction pathway, is essential in numerous intracellular processes, including growth, proliferation, differentiation, and immune-mediated responses. MAPK signaling pathways, composed of diverse kinases among which p38 MAPK, ERK, and c-Jun-activated kinase (JNK) are the three most important ones, are activated by phosphorylations of threonine and tyrosine residues mediated by upstream MAPK kinases (MKK). MAPK pathways are of great importance for the regulation of cell inflammatory immune responses (Yang et al., 2013). Studies have shown that IL-12 secretion in *T. gondii* infected macrophages is stimulated by the activation of host p38α MAPK via MKK-independent autophosphorylation (Kim et al., 2005) or TRAF6-dependent phosphorylation (Mason et al., 2004). *T. gondii* GRA24, a dense granule protein, can trigger nuclear translocation and autophosphorylation of host p38 MAPK, which correlates with the synthesis of IL-12 (Braun et al., 2013). Toll-like receptors (TLRs) are pattern-recognition receptors which, after binding with ligands, stimulate pro-inflammatory activity against pathogen infection. Signaling through TLRs has been demonstrated to be important in the host defense against *T. gondii* (Yarovinsky, 2008). In *T. gondii* infection, the profilin-like ligand can be recognized by host TLR11 and TLR12 to regulate IL-12 synthesis in macrophages and DC cells (Yarovinsky et al., 2005; Plattner et al., 2008; Koblansky et al., 2013; Hedhli et al., 2016). However, in *N*. *caninum* infection, the regulation mechanism of inflammatory cytokine responses has not been fully elucidated. Recent studies show that *N. caninum* activates p38 MAPK to down-regulate the host’s innate immune responses, and blockade of p38 MAPK results in an amplified production of IL-12p40 in macrophages against the infection (Mota et al., 2016).

In order to clarify the underlying mechanism of pro-inflammatory cytokine IL-12 production by *N. caninum* infected macrophages, the roles of TLR11 and MAPK signaling were examined in the present study. The results presented here implicated that TLR11 and MEK/ERK signaling were involved in IL-12p40 expression in *N. caninum* infected peritoneal macrophages.

## Materials and Methods

### Animals and Ethics Statement

Female C57BL/6 mice (5 to 6 weeks) free of pathogen were obtained from the Experimental Animal Center of Jilin University. Animal experiments were performed in accordance with the recommendations in the Guide for the Care and Use of Laboratory Animals of the National Institutes of Health. The protocol was approved by the Animal Care and Use Committee of Jilin University.

### Preparation and Identification of Peritoneal Macrophages

C57BL/6 mice were injected with 4 μl Fluid Thioglycollate medium (Becton, Dickinson and Company, United States) intraperitoneally, and the mouse peritoneal macrophages (PMϕ) were collected 4 days later under aseptic conditions. After rinsing twice with sterile PBS by centrifugation at 850 ×*g* for 10 min at 4°C, PMϕ (1 × 10^5^/ml) were cultured in 6-WP (Hyclone, Rochester, NY, United States) in DMEM (high glucose) supplemented with 10% heat-inactivated fetal bovine serum (FBS) and antibiotic–antimycotic reagents (all from Life Technologies, Carlsbad, CA, United States) at 37°C with 5% CO_2_. Non-adhered cells were carefully washed away and fresh medium was added at 2 h. Collected macrophages were confirmed using a mAb against mouse CD11b (1/500; Abcam, United States) by flow cytometry and immunofluorescence assay. For Flow cytometry, cells were collected by treatment with Trypsin (0.25%) supplemented with 0.1% EDTA-2Na (Life Technologies). For IFA, monolayers were fixed with 4% paraformaldehyde for 15 min and blocked in 5% w/v bovine serum albumin (BSA) for 1 h at 37°C. For CD11b staining, cells were incubated with the primary antibody in PBST containing 3% BSA for 1 h at 37°C, followed by incubation with the Alexa fluorescent 488-conjugated goat anti-rabbit IgG (1/500; Proteintech, United States) as the secondary antibody for 1 h at room temperature in the dark in PBST with 3% BSA. Stained cells were either analyzed by the Attune NxT Flow cytometer (Thermo Fisher Scientific, United States) or observed under a FV1000 confocal microscopy (Olympus Co., Japan) after counter stained with DAPI. Specificity of staining was determined by incubating monolayers with secondary antibody alone.

### Cell Transfection

PMϕ (1.5 × 10^5^) in 6-WP were transiently transfected with control siRNA or siRNA against mouse Myd88 (Cell Signaling, United States) using FuGENE HD transfection reagent (Roche Applied Science, Indianapolis, IN, United States) according to the manufacturer’s instructions. Gene silencing was confirmed by Western blot at 36 h after transfection.

### Parasites Culture and Purification

Tachyzoites of *N. caninum*-1 strain were stored at our laboratory. Vero cells were cultured in DMEM supplemented with 10% FBS. Cells were infected with *Nc*-1 tachyzoites and cultured in DMEM with 2% FBS for 3–5 days at 37°C and 5% CO_2_. After spontaneous cell rupture, cell debris mixed with tachyzoites was harvested. After centrifugation, the pellet was resuspended in cold DMEM medium and passed through a 26-gauge needle (Millipore, Billerica, MA, United States). The obtained mixture was slowly layered on a 40% Percoll solution (GE Healthcare, United States) in DMEM without FBS and separated by centrifugation at 850 ×*g* in a horizontal centrifuge for 30 min. The fraction containing tachyzoites at the bottom of the tube was collected and washed in DMEM without FBS by centrifugation at 850 ×*g* at 4°C for 10 min. The final tachyzoite pellet was resuspended in DMEM without FBS.

### Tachyzoite Infection Protocols

PMϕ were sterilely obtained and cultured in 6-WP (Hyclone, Rochester, NY, United States) in DMEM with 10% FBS. Cell monolayers were washed twice with PBS and the culture medium was replaced by DMEM F-12 with 1% FBS before infection. Cells were infected with *Nc*-1 tachyzoites at a multiplicity of infection (MOI) of 10 or treated with lipopolysaccharide (LPS) from *E. coli* at 50 ng/ml (Sigma–Aldrich, St. Louis, MO, United States) for indicated times, respectively. In addition, some sets of monolayers were infected with tachyzoites with MOIs of 0, 2.5, 5, and 10 for 1 h, respectively. At different time points post-treatment, culture supernatants were removed from macrophage monolayers for ELISA, or cell lysates were prepared for Western blotting.

For treatment with UO126 or PD98059 (Selleck Chemicals, United States), which are specific kinase inhibitors against ERK and ERK signaling upstream MEK, respectively, PMϕ were treated with tachyzoites (MOI = 10) or LPS (50 ng/ml) in the presence or absent of UO126/PD98059 at different concentrations (5–20 μM) under conditions discussed in the results section; for pre-treatment with UO126/PD98059, PMϕ were incubated with the inhibitor (20 μM) or DMSO for 1 h and after washing with sterile PBS, tachyzoites or LPS were added; for treatment with monoclonal antibodies against TLR11 (ab47097, Abcam, United States) (Chen et al., 2014), PMϕ were incubated with the antibodies at the concentration of 3 μg/ml or same amount of homologous negative serum for 2 h at 37°C before tachyzoites (MOI = 10) were added to the cells for 1, 24, and 48 h, respectively; for treatment with siRNAs, PMϕ were inoculated with tachyzoites (MOI = 10) for 1 and 24 h, respectively, after transfection with Myd88 siRNA or control siRNA for 36 h. After different treatments, supernatants and cells were collected for ELISA and Western blotting.

### Western Blotting

After treatments, cells were washed twice with PBS and cells were collected by centrifugation. The pellet was resuspended in lysis buffer supplemented with protease and phosphatase inhibitors (Sangon Biotech Co., Shanghai, China) and lysed on ice for 15 min. Cell lysates were collected following centrifugation at 10,000 ×*g* for 15 min at 4°C. Supernatants were mixed with loading buffer, boiled for 5 min, and centrifuged at 10,000 ×*g* for 5 min. Protein samples of the same amount were electrophoresed on 12% SDS-PAGE gels (Bio-Rad Laboratories, Inc., United States) and transferred to nitrocellulose membranes (Pall Life Sciences, United States). Membranes were blocked in 5% skim milk (w/v) in TBST for 2 h at room temperature. Membranes were incubated with the specific Phospho-ERK (Thr202/Tyr204, 197G2, 44/42 kDa), Phospho-MEK (Ser217/221, 41G9, 43/44 kDa), ERK (137F5, 44/42 kDa), MEK (D1A5, 43/44 kDa) (1/1,000; all from Cell Signaling) and TLR11 (ab47097, 95 kDa, 1/500; Abcam) rabbit mAb overnight at 4°C followed by incubation with horseradish peroxidase-conjugated goat anti-rabbit IgG (1/2,000; Proteintech, United States) for 1 h at 37°C in TBST. Protein bands were visualized by using an enhanced chemiluminescence kit (Proteintech Group Inc., United States) and detected using the ChemiScope series 5300 (Clinx Science Instruments Co., Ltd, Shanghai, China) according to the manufacturer’s instructions. Beta-actin was used as the internal control protein. The intensities of phospho-ERK or MEK were calculated using ImageJ (NIH) and normalized against total ERK or MEK.

### IL-12p40 Detection by ELISA

To measure cytokine IL-12 secreted by PMϕ, cell culture supernatants were collected at different times post-treatment as described above. Secretion levels of IL-12 (p40) were assessed using an ELISA kit (eBioscience, Inc., San Diego, CA, United States) following the manufacturer’s instructions. Samples were analyzed in triplicates in 96 well plates, which had been pre-coated with capture antibody. Plates were developed using HRP substrate solutions and plates were read at 450 nm on a Powerwave 200 spectrophotometer (Bio-Tek, Winooski, VT, United States). The cytokine concentration in each sample was extrapolated from a standard curve generated from the measured absorbance obtained from standards supplied with the kit.

### Statistics

Results were analyzed for statistical significance with Student’s *t*-test and one-way ANOVA by SPSS 19.0 software. Data are presented as the mean ± standard deviation. Differences were considered significant compared to control at a *P*-value of < 0.05 (^∗^*P* < 0.05 and ^∗∗^*P* < 0.01).

## Results

### *N. caninum* Infection Induces ERK1/2 Activation in PMϕ

**FIGURE 1:**
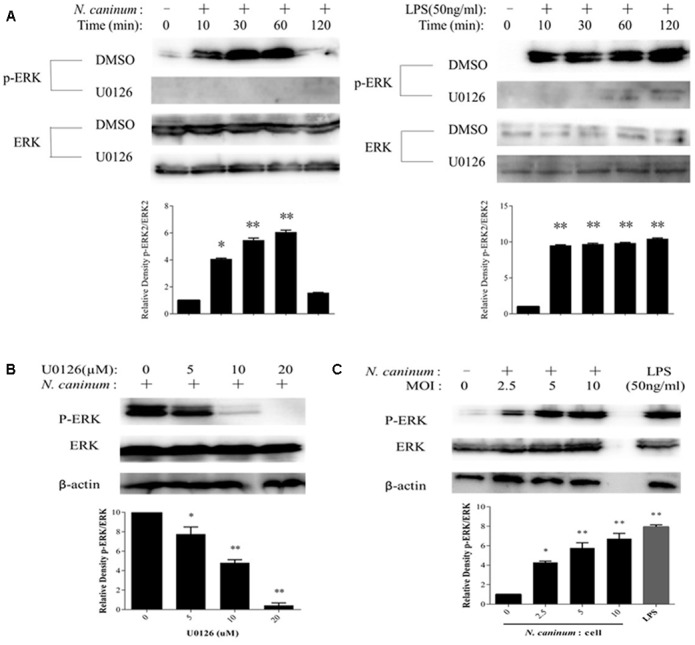
*N. caninum* infection induces ERK1/2 activation of PMϕ. **(A)** After incubated with U0126 (20 μM) or vehicles for 1 h, PMϕ were treated with freshly purified *N. caninum*-1 tachyzoites (MOI = 10) or LPS (50 ng/ml) for different times at 37°C. **(B)** After incubated with U0126 (5–20 μM) or DMSO for 1 h, PMϕ were infected with *NC*-1 tachyzoites for 1 h at 37°C. **(C)** PMϕ were infected with tachyzoites with MOI of 0, 2.5, 5, and 10, respectively, or treated with LPS (50 ng/ml) for 1 h at 37°C. After treatments, cell lysates were prepared for determination of ERK1/2 phosphorylation at Thr202/Tyr204 and total ERK1/2 by Western blotting. The intensities of phospho-ERK were calculated using ImageJ (NIH) and normalized against total ERK. Results are representative of three independent experiments. Data are expressed as the mean ± SEM. ^∗^*P* < 0.05; ^∗∗^*P* < 0.01.

Identified PMϕ (Supplementary Figure 1) were infected with freshly purified tachyzoites or treated with LPS (50 ng/ml), followed by determination of ERK1/2 phosphorylation at Thr202/Tyr204 by Western blotting. The results showed the rapid activation of ERK1/2 in response to *N. caninum* infection in PMϕ. ERK 1/2 was phosphorylated within 10 min of infection and reached a plateau at 1 h, then quickly dropped as indicated in **Figure 1A**. On the other hand, ERK1/2 was phosphorylated and maintained its higher levels during the whole experiment period when treated with LPS (**Figure 1A**). Additionally, the activation of ERK1/2 by *N. caninum* showed a parasite load-dependent response as indicated in **Figure 1C**. Thus, *N. caninum* caused ERK1/2 activation in PMϕ.

To evaluate the inhibitory effect of U0126, (a chemical inhibitor for ERK) (DeSilva et al., 1998), on ERK1/2 activation by *N. caninum*, PMϕ were pre-treated with DMSO or U0126 for 1 h, followed by infection with tachyzoites or not. The results of Western blotting showed that ERK1/2 phosphorylation at Thr202/Tyr204 was largely reduced when treated with U0126 (**Figure 1A**). Inhibition of ERK1/2 phosphorylation by the inhibitor presented a dose-dependent response as indicated in **Figure 1B**. U0126 at 20 μM could completely abolish the phosphorylation. Hence, U0126 inhibit ERK1/2 activation in PMϕ induced by *N*. *caninum* infection.

### ERK1/2 Activation Induced by *N*. *caninum* Is Mediated by MEK1/2 Activation in PMϕ

**FIGURE 2:**
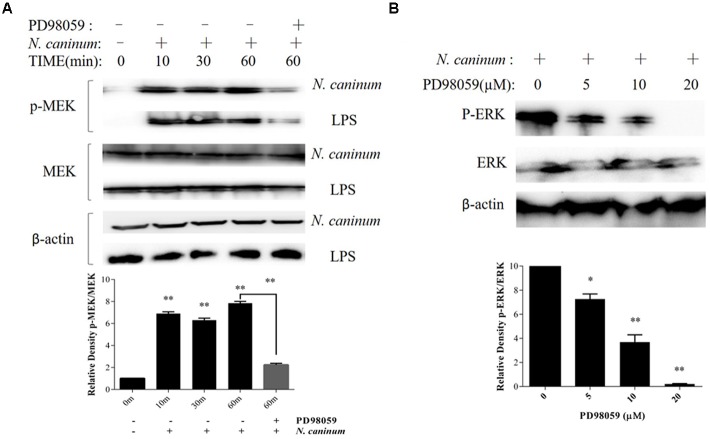
*N. caninum* infection induces ERK-MEK pathway activation of PMϕ. **(A)** PMϕ were infected with freshly purified *N. caninum*-1 tachyzoites (MOI = 10) or treated with LPS (50 ng/ml) for different times at 37°C. For the last lane, cell monolayers were treated with tachyzoites or LPS in the presence of PD98059 (20 μM) for 1 h. After treatments, cells were collected for determination of MEK1/2 phosphorylation at Ser217/221 and total MEK by Western blot. **(B)** After incubated with PD98059 (5–20 μM) or DMSO for 1 h, PMϕ were infected with tachyzoites at a MOI of 10 for 1 h at 37°C, followed by determination of ERK1/2 phosphorylation at Thr202/Tyr204 and total ERK. The intensities of phospho-ERK or MEK were calculated using ImageJ (NIH) and normalized against total ERK or MEK. Data represent the mean of 3 independent experiments ± SEM. ^∗^*P* < 0.05; ^∗∗^*P* < 0.01.

To evaluate the effect of MEK1/2 on ERK1/2 activation by *N. caninum*, PMϕ were pre-incubated with DMSO or PD98059 (20 μM) for 1 h, followed by infection with tachyzoites. The results showed that *N. caninum* caused MEK1/2 activation in PMϕ (**Figure 2A**), which were coincided with the phosphorylation of ERK1/2 during infection. Moreover, MEK1/2 (**Figure 2A**) activation and ERK1/2 (**Figure 2B**) phosphorylation were both significantly reduced when treated with PD98059, a specific inhibitor for MEK1/2. The inhibition of ERK1/2 phosphorylation by PD98059 showed a dose-dependent response. PD98059 at 20 μM could completely inhibit the ERK1/2 phosphorylation as indicated in **Figure 2B**. Thus, *N*. *caninum* induced ERK1/2 phosphorylation in PMϕ was triggered by the activation of upstream MEK1/2.

### *N. caninum* Infection Induces Cytokine IL-12p40 Production by PMϕ

**FIGURE 3:**
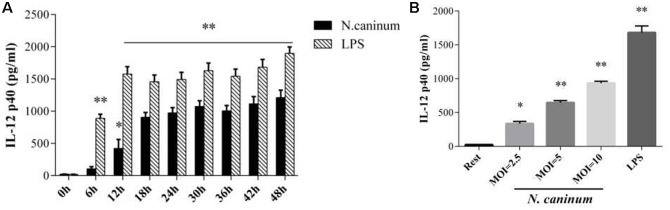
*N. caninum* infection induces cytokine IL-12 production by PMϕ. **(A)** PMϕ monolayers were infected with *N. caninum*-1 tachyzoites at a MOI of 10 and treated with 50 ng/ml LPS in DMEM with 1% FBS at 37°C for 0–48 h, respectively. **(B)** PMϕ were treated with tachyzoites at the MOI of 2.5, 5, 10, or LPS (50 ng/ml) for 24 h, respectively. After treatments, cell culture supernatant were collected for ELISA to measure the secretion levels of IL-12. Samples were applied in triplicates. Results represent the mean of three independent experiments ± SEM. ^∗^*P* < 0.05; ^∗∗^*P* < 0.01.

To assess the inflammatory IL-12p40 responses produced by macrophages infected with *N. caninum*, PMϕ were infected with tachyzoites or treated with LPS (50 ng/ml), and followed by determination of secretion levels of the cytokine. ELISA results showed that IL-12p40 was increased when treated with both *N. caninum* and LPS. After infection with the parasites, IL-12p40 was significantly increased compared to control at 12 h (*p* < 0.05, **Figure 3A**). IL-12p40 reached the first plateau within 24–30 h of infection and then sustained at high level during the rest of infection time. On the other hand, IL-12p40 was rapidly upregulated (*p* < 0.05 at 6 h) to reach the first plateau at just 12 h (*p* < 0.01) and maintained at high level throughout the experiment as indicated in **Figure 3A**. Tachyzoites stimulated IL-12p40 exocytosis showed a parasite dose-dependent response (**Figure 3B**). Thus, *N. caninum* infection induced IL-12p40 production by infected macrophages.

### IL-12p40 Production in Macrophages Infected with *N*. *caninum* Is Induced by MEK-ERK Activation

**FIGURE 4:**
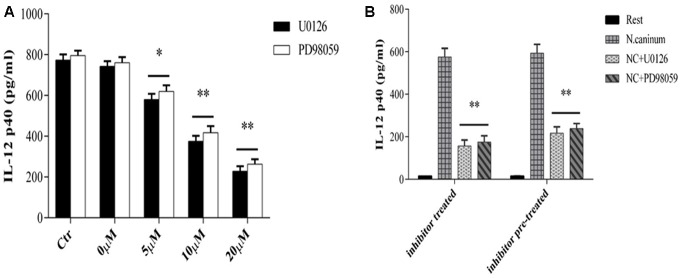
U0126 and PD98059 inhibit IL-12 production by macrophages infected with *N. caninum.* **(A)** PMϕ were infected with *N. caninum*-1 tachyzoites at a MOI of 10 in the presence or absence (Ctr) of UO126 and PD98059 (0–20ìM, 0ìM was treated with same amount of DMSO), respectively, in DMEM with 1% FBS at 37°C for 24 h. **(B)** PMϕ were incubated with U0126 and PD98059 with 20 μM for 1 h, respectively. Ctr was incubated with same amount of DMSO. Then after the inhibitor or DMSO was washed off (pre-treatment) or not (treatment), PMϕ were infected with tachyzoites at a MOI of 10 for 24 h at 37°C. Samples were applied in triplicates for ELISA assay and data are expressed as the mean of three independent experiments ± SEM. ^∗^*P* < 0.05; ^∗∗^*P* < 0.01.

Next, we assessed the role of ERK signaling pathway in IL-12 immune responses induced by *N. caninum* infection though examining the effect of inhibition ERK1/2 and MEK1/2 signaling on cytokine secretion of infected macrophages. PMϕ were treated with or without U0126/PD98059 and followed by determination of secretion levels of cytokine IL-12p40 at 24 h of infection. The results (**Figure 4A**) showed that U0126/PD98059 caused marked decrease in the concentrations of IL-12p40 at 24 h (*p* < 0.05) and, especially with U0126. Moreover, the inhibition of IL-12p40 production by U0126/PD98059 showed a dose-dependent response. U0126 and PD98059 at concentrations of 20 μM caused decreases of 72.8 and 68.8% in the secretion, respectively (*p* < 0.01, **Figure 4B**). What’ more, when the inhibitor was removed (pre-treatment, 20 μM), the inhibition effect in the cytokine secretion could not be completely reversed (*p* > 0.05 when compared to inhibitor treatment, **Figure 4B**). These results suggested that activation of host cell MEK-ERK pathway by *N. caninum* stimulated macrophage IL-12p40 production and host MEK-ERK signaling played an active and important role in IL-12 production stimulated by the parasite.

### ERK1/2 Activation and IL-12p40 Production Induced by *N*. *caninum* Are Mediated by TLR11 in PMϕ

**FIGURE 5:**
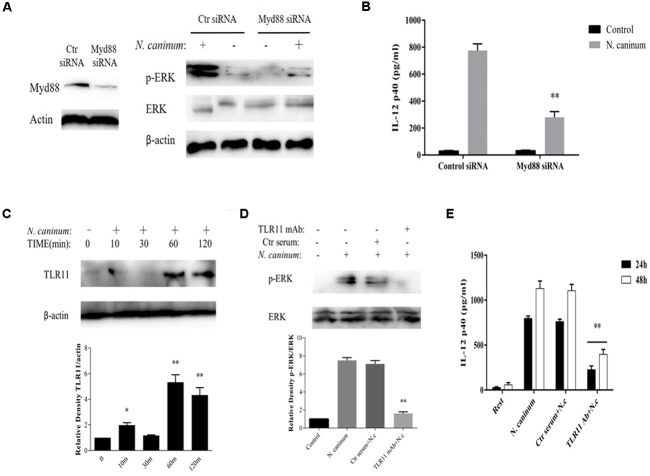
IL-12 production by macrophages infected with *N. caninum* is TLR11-dependent. PMϕ were transfected with Myd88 siRNA or control siRNA before infection. After treatments, cell lysates were prepare at 1 h for determination of ERK1/2 activation by Western blot **(A)** and culture supernatants were collected at 24 h for ELISA to measure the IL-12 secretion **(B)**. **(C)** PMϕ were infected with freshly purified *N. caninum*-1 tachyzoites at a MOI of 10 for different times at 37°C. **(D)** PMϕ were infected with tachyzoites for 1 h after pre-treatment with antibodies specifically against mouse TLR11 (3 μg/ml) or homologous negative serum both for 2 h at 37°C. **(E)** After incubation with mAb against TLR11 or same amount of control serum, PMϕ were infected with tachyzoites for 24 and 48 h. β-actin was used as the internal control. The intensities of phospho-ERK were calculated using ImageJ (NIH) and normalized against total ERK. Results represent the mean of three independent experiments ± SEM. ^∗^*P* < 0.05; ^∗∗^*P* < 0.01.

To examine the potential role of TLR signaling in ERK pathway activation and IL-12 production by *N. caninum*, PMϕ cells were either treated with a neutralizing antibody against mouse TLR11 or transfected with a siRNA targeted against Myd88 knockdown, respectively. The results from Western blotting and cytokine ELISA showed that transfection with siRNA against mouse Myd88 caused dramatic decrease in the phosphorylation of ERK1/2 at 1 h (*p* < 0.01, **Figure 5A**) and production of IL-12p40 after *N. caninum* infection (Secretion of IL-12p40 was reduced by 63.9% at 24 h, *p* < 0.01, **Figure 5B**), suggesting that Myd88 worked at the upstream of ERK signaling to regulate IL-12p40 production in PMϕ after *N. caninum* infection.

In addition, *N. caninum* infection induced the upregulation of TLR11 in macrophages (**Figure 5C**) which reached a plateau at 1 h (*p* < 0.01). Furthermore, after incubation with mAb against TLR11, phosphorylation of ERK1/2 1 h post-infection were dramatically inhibited (*p* < 0.01, **Figure 5D**) and extracellular secretion of IL-12p40 was reduced by 72.5% at 24 h and 64.2% at 48 h (*p* < 0.01, **Figure 5E**), while incubation with control serum could not cause inhibition (*p* > 0.05). This indicated that the ERK signaling is activated via TLR11 which in turn upregulated the synthesis of IL-12p40 in peritoneal macrophages in response to *N*. *caninum*.

## Discussion

*Neospora caninum* is a primary pathogen and one of the most efficiently transplacentally transmitted organisms in cattle and dogs (Reichel and Ellis, 2008). Livestock infection with *N. caninum* generally showed few clinical symptoms. *N. caninum* has evolved to reach a balance between triggering and avoiding the host immune response as a result of the interaction between the host and the parasite (Innes et al., 2005; Dubey et al., 2007).

Host factors including cytokines which may contribute to pathogenesis and host resistance of *N. caninum* infection have not yet been fully established. Studies on factors that regulate the pro-inflammatory immune response against *N. caninum* are limited. The present results showed that *N*. *caninum* infection caused IL-12p40 exocytosis in peritoneal macrophage was induced by activation of MEK-ERK signaling pathway. Blocking MEK-ERK signaling by UO126 and PD98059 effectively inhibited the IL-12p40 secretion from *N*. *caninum* infection. In addition, when the inhibitor against ERK or MEK was removed, the cytokine secretion was only partly restored, suggesting the irreversibility of these inhibitors on ERK1/2 or MEK1/2 homologs. Pre-treatments of peritoneal macrophages with the inhibitors were enough to significantly suppress IL-12p40 production, suggesting that the inhibition effect of UO126 and PD98059 is specifically directed to host cells. MAPK homologs in Apicomplexan protozoa have been identified (Lacey et al., 2007; Brown et al., 2014). Therefore, inhibitors against mammalian MAPK might be able to act on MAP kinases in the parasite. The present results indicated that host MEK and ERK were the main kinases involved in IL-12p40 production in *N. caninum* challenged peritoneal macrophages, while parasite MAP kinases did not seem to be involved in this process of MEK-ERK activation-dependent IL-12p40 production.

Host cell invasion by *N*. *caninum* elicits the host immune responses, including humoral immunity and cellular immunity, to resist the infection. Cell-mediated immunity (CMI) is believed to play a major role in protection against infection from obligate intracellular parasites, such as *T. gondii* and *N. caninum* (Baszler et al., 1999; Innes et al., 2002). Secretion of pro-inflammatory cytokines during *N*. *caninum* infection, such as IL-12, TNF-α, and IFN-γ, is necessary for control of parasite infection by host immune system (Khan et al., 1997; Mineo et al., 2009; Teixeira et al., 2010). IL-12, a well known inducer of IFN-γ production, is important for the generation of Th1-type responses. It has been reported that IFN-γ production was largely abrogated in IL-12/IL23 p40-deficient mice challenged with *N. caninum* (Teixeira et al., 2016). Studies have shown that host IL-12 developed a Th1-type immune response which is necessary for host control of parasite infection (Nishikawa et al., 2002; Williams and Trees, 2006). IL-12 stimulated IFN-γ synthesis by natural killer (NK) cells and T lymphocytes controls intracellular replication of *Toxoplasma* (Sher et al., 2003; Suzuki et al., 2012). Recombinant IL-12 induces IFN-γ-dependent protection against malaria (Sedegah et al., 1994). Although dendritic cells are main IL-12 producers in *N. caninum* or *T. gondii* challenged animals, macrophages are the most important phagocytic cells in mammals which play critical roles in detection and elimination of pathogens. To understand how IL-12 production is manipulated in macrophages during *N*. *caninum* infection is essential for parasite control. Macrophage produces little IL-12 at the initial 24 h after *T. gondii* infection, which is resumed after that (Butcher et al., 2001). Our present results indicated that IL-12p40 production was very low within 12 h of infection, but was significantly enhanced (*p* < 0.05) after that when compared to control, and then sustained at high level during the rest of infection time in PMϕ. Low levels of IL-12 in early infection might be important for the parasite to not only survive and establish chronic infection, but also not to kill the host cells, which was in accordance with previous reports in *T. gondii* infection (Denkers et al., 2003). In our study, bacterial LPS was used as a positive control. The present results showed that the activation of ERK signaling and IL-12p40 production induced by *N. caninum* were different from that induced by LPS.

The present study also showed that *N*. *caninum* infection upgraded TLR11, and blockade of TLR11 by a specific neutralizing antibody notably suppressed ERK activation and IL-12p40 exocytosis triggered by *N*. *caninum*. IL-12 production induced by Apicomplexan parasites is regulated by various signal transduction pathways. In *T. gondii* infection, previous studies have shown that p38 MAPK phosphorylation triggers macrophage IL-12 production (Mason et al., 2004; Kim et al., 2005). Inhibition of NF-κB translocation may be related to delayed IL-12 production in *Toxoplasma*-infected macrophages, while NF-κB independent pathways of IL-12 production are essential for the control of *T. gondii* infection (Butcher et al., 2001; Denkers et al., 2004). The profilin-like ligand potently stimulates TLR11 and regulates the production of IL-12, which is necessary for the protective IFN-γ response (Plattner et al., 2008). In *Leishmania amazonensis* infection, activation of PI3K/Akt signaling suppresses IL-12 production by bone marrow derived macrophages (Ruhland and Kima, 2009). Current results suggested that TLR11-mediated activation of ERK signaling was involved in eliciting IL-12p40 production triggered by *N. caninum* infection, which implied that *N. caninum* infection induces positive signaling that mediates IL-12 production. While other studies indicated that *N. caninum* evades the host’s IL-12 pro-inflammatory immune response by activation of p38 MAPK signaling pathway, and inhibition of p38 enhances IL-12 production which results in decreased parasite load and increased host macrophage survival (Mota et al., 2016). Our results provided evidence that MEK-ERK signaling pathway played an important role in IL-12p40 secretion in macrophages infected with *N. caninum*. These studies demonstrate that MAP kinases differentially regulate IL-12 production in macrophages in response to *N. caninum*. Activation of p38 by parasite is mediated by a mechanism which depends on G protein-coupled receptor (GPCR)/PI3 kinase/AKT signaling pathways. Our present results indicated that blockade of TLR11-ERK pathway led to decreased IL-12p40 production which is MEK and Myd88-dependent. It is worth noting that bone marrow-derived macrophages (BMDMs) were used in the experiments referenced above, while peritoneal macrophages were used in our studies. In addition, downregulation of IL-12 production via p38 MAPK activation during *Neospora* infection are independent of C-C chemokine receptor 5 (CCR5) and classical transcription factors such as NF-κB, AP-1, mTOR, and JAK2. Transcription factors that are key players in pro-inflammatory response may be involved with IL-12p40 production induced by *Neospora*-activated MEK-ERK, which needs further studies.

TLR4 (LPS) or TLR2 (glycosylphosphatidylinositol anchors, GPI) mediated microbial signaling triggers MAPK and NF-κB signaling cascades that lead to pro-inflammatory response (Yarovinsky and Sher, 2006). Research showed that TLR2^-/-^ mice presented higher parasite burden than wild-type mice at acute and chronic stages of infection by *N. caninum* with diminished IFN-γ/IL-10 ratio (Mineo et al., 2010). The importance of TLRs in innate resistance to *T. gondii* infection has been demonstrated (Mun et al., 2003; Andrade et al., 2013). It has been reported that MyD88^-/-^ mice are acutely susceptible to *Neospora* and *Toxoplasma* infections with defective IL-12 production (Scanga et al., 2002; Mineo et al., 2009). The present results using siRNA against Myd88 also suggested that Myd88 was involved in *N. caninum* induced ERK signaling transduction and IL-12p40 production mediated by TLR11. TLR11 has been demonstrated to be important in mediating IL-12 response in *T. gondii* infection. Studies have showed that *T. gondii* profilin-like proteins stimulate IL-12 response through TLR11/TLR12 (Yarovinsky et al., 2005; Koblansky et al., 2013; Hedhli et al., 2016). In the present study, we determined that IL-12p40 production during *N*. *caninum* infection was also TLR11 dependent, which might be mediated by the interaction between the tachyzoite antigen molecules such as profilin and TLR11 on the surface of host cells, suggesting that *N. caninum* could also stimulate TLR11 to upregulate the host’s innate immune responses. Results from the present study suggested that TLR-11 mediated ERK1/2 pathway appeared to be an important mechanism for IL-12p40 production in PMϕ infected with *N. caninum*. However, TLR11 is not expressed in all *N. caninum* hosts, such as bovines, and this may limit the reach of the observed findings. Also, In view of the fact that IL-12p40 is also a component of IL-23, the present results could not rule out the involvement of IL-23, which needs to be further studied.

Taking together, the study presented here demonstrated, for the first time, that *N*. *caninum* infection triggered upregulation of TLR11 and subsequent activation of MEK-ERK signaling pathway which in turn induced IL-12p40 production. These new findings will help to further our understanding of parasite-host interaction and the protective immune response by the host against *N*. *caninum* infection. For future studies, TLR11-knockout mice will be needed to elucidate the complex signal network, and to determine the ligand molecules involved in TLR11 recognition in this process as well as the precise function of ERK signaling in *N*. *caninum* infection and host resistance.

## Author Contributions

JL and XZ conceived and designed the study. XJ, TZ, and MZ performed the experiments. XJ drafted the manuscript. PG analyzed the data. JL and GL critically revised the paper. All authors read and approved the final version of the manuscript.

## Conflict of Interest Statement

The authors declare that the research was conducted in the absence of any commercial or financial relationships that could be construed as a potential conflict of interest.
