# Supplementary material for: Activation of ERK Signaling via TLR11 Induces IL-12p40 Production in Peritoneal Macrophages Challenged by Neospora caninum
Source: Front Microbiol. 2017 Jul 26;8:1393. doi: 10.3389/fmicb.2017.01393 (PMC5527353; doi:10.3389/fmicb.2017.01393)
Supplement: Supplementary file 1 [file Data_Sheet_1.DOCX]

Supplementary Material

**Activation of ERK Signaling via TLR11 Induces IL-12p40 Production in Peritoneal Macrophages Challenged by** ***Neospora caninum***

**Xiaoxia Jin, Pengtao Gong, Xichen Zhang, Guojiang Li, Tao Zhu, Mengge Zhang,** **Jianhua Li***

*** Correspondence:** Jianhua Li: Jianhuali7207@163.com (J. Li)

# Supplementary Data

**Preparations and Identification of Peritoneal Macrophages**

PMϕ were identified by detecting the expression of CD11b on the surface of macrophages. The results from Flow cytometry and Immuno-fluorescence staining suggested that CD11b was shown in the isolated mouse cells, as shown in Figure 1A and 1B, indicating our obtained cells were peritoneal macrophages. About 3-5 × 10^5^ macrophages were obtained from each mouse. The purity of isolated PMϕ is above 96%.

# Supplementary Figures and Tables

## Supplementary Figures


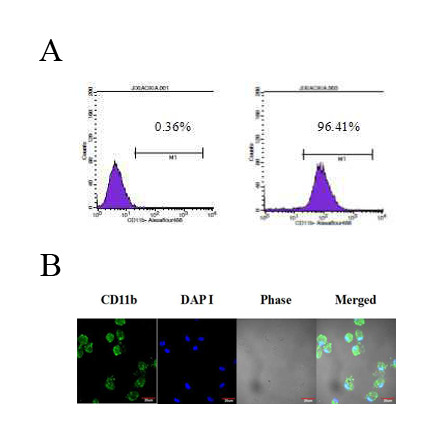


**Supplementary Figure 1. Identification of Peritoneal Macrophages.**

Obtained peritoneal macrophages were identified using a specific antibody against mouse CD11b by Flow cytometry (A, Specificity of staining was determined by incubating monolayers with secondary antibody alone, the left lane) and Immunofluorescence assay (B). Results are representative of 2 independent experiments.
